# The logic of transgenerational inheritance in a nematode worm

**DOI:** 10.1016/j.isci.2026.117401

**Published:** 2026-09-03

**Authors:** Shiela Pearl Quiobe, Ata Kalirad, Ralf J. Sommer

**Affiliations:** 1 Department for Integrative Evolutionary Biology, Max Planck Institute for Biology Tübingen, 72076 Tübingen, Germany

**Keywords:** transgenerational epigenetic inheritance, dynamics of non-genetic inheritance, cannibalism, developmental plasticity, nematode

## Abstract

Epigenetic inheritance of environmentally induced phenotypes across generations has been observed in multiple taxa. However, the dynamics of non-genetic inheritance as a consequence of environmental variability remains poorly understood. Here, we take advantage of the transgenerational epigenetic inheritance (TEI) of an environmentally induced cannibalistic trait in the nematode *Pristionchus pacificus*. Using alternative environmental induction and superinduction experiments, we illustrate how environmental variability regimes influence TEI and uncover the dosage-dependent nature of the trigger molecule—vitamin B12—on the onset of TEI. Our study provides a unique example of the dynamics of TEI of an ecologically relevant trait as a consequence of environmental variation.

## Highlights

• TEI of predatory mouth form can be constant, acclimating, or sensitizing
• TEI is history-independent for short intervals, sensitizing over long ones
• Higher vitamin B12 shifts TEI: earlier onset, slower morph-memory decay
• Theory and experiments reveal mixed TEI dynamics in a nematode trait

## Graphical abstract

**Figure:**
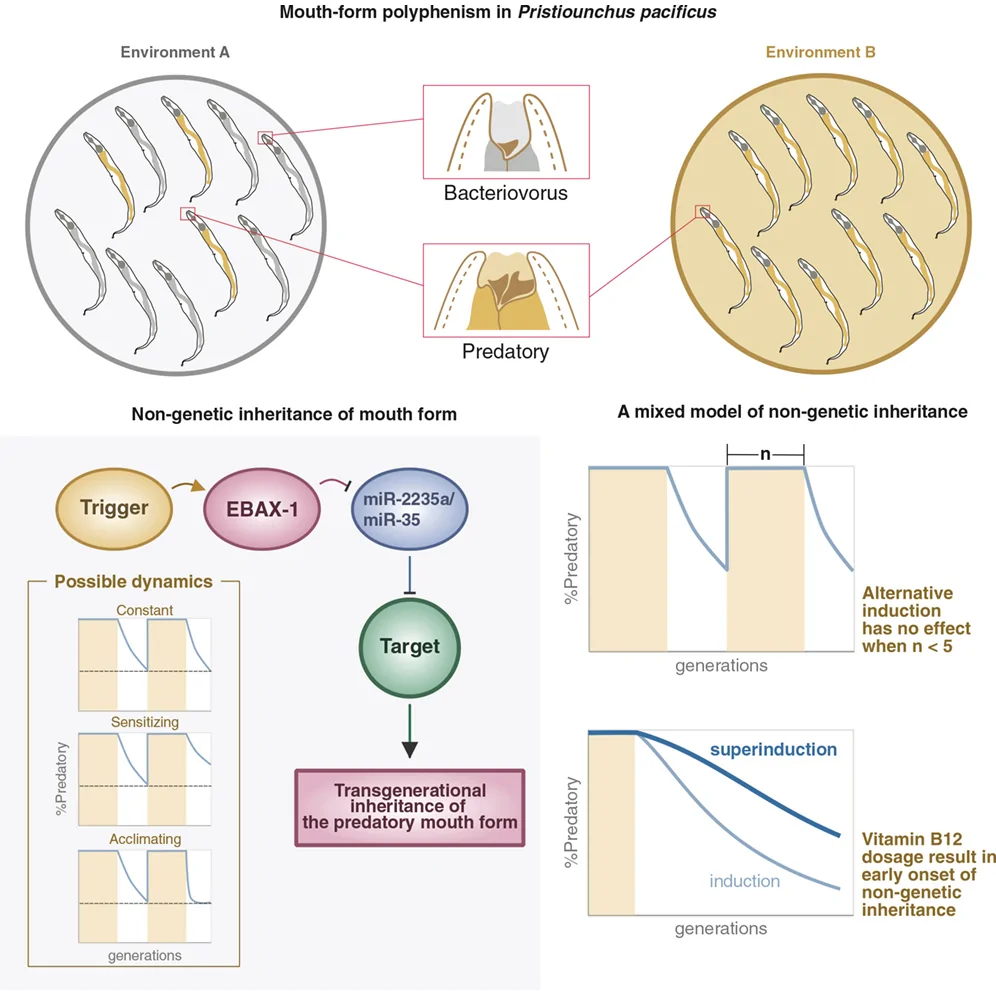

## Teaser

Zoology; Evolutionary biology; Evolutionary developmental biology

## Introduction

Epigenetic inheritance has been reported in a plethora of organisms, ranging from inheritance of odor sensitivity in mice and longevity in *Caenorhabditis elegans* to famine-induced epigenetic changes in humans.^1^^,^^2^^,^^3^^,^^4^ Despite these examples, the importance of epigenetic inheritance in evolution remains disputed.^5^^,^^6^^,^^7^^,^^8^ Importantly, the inheritance of epigenetic changes across three generations or more following the exposure to an environmental cue—referred to as transgenerational epigenetic inheritance (TEI)^9^^,^^10^—is thought to provide evolutionary benefits in fluctuating environments. However, there are few experimental studies of the dynamics of TEI (e.g., Houri-Zeevi et al.^11^) and, to our knowledge, there have been no case studies which investigated the pattern of TEI in response to environmental variability for an ecologically-relevant phenotype.

**Figure 1:**
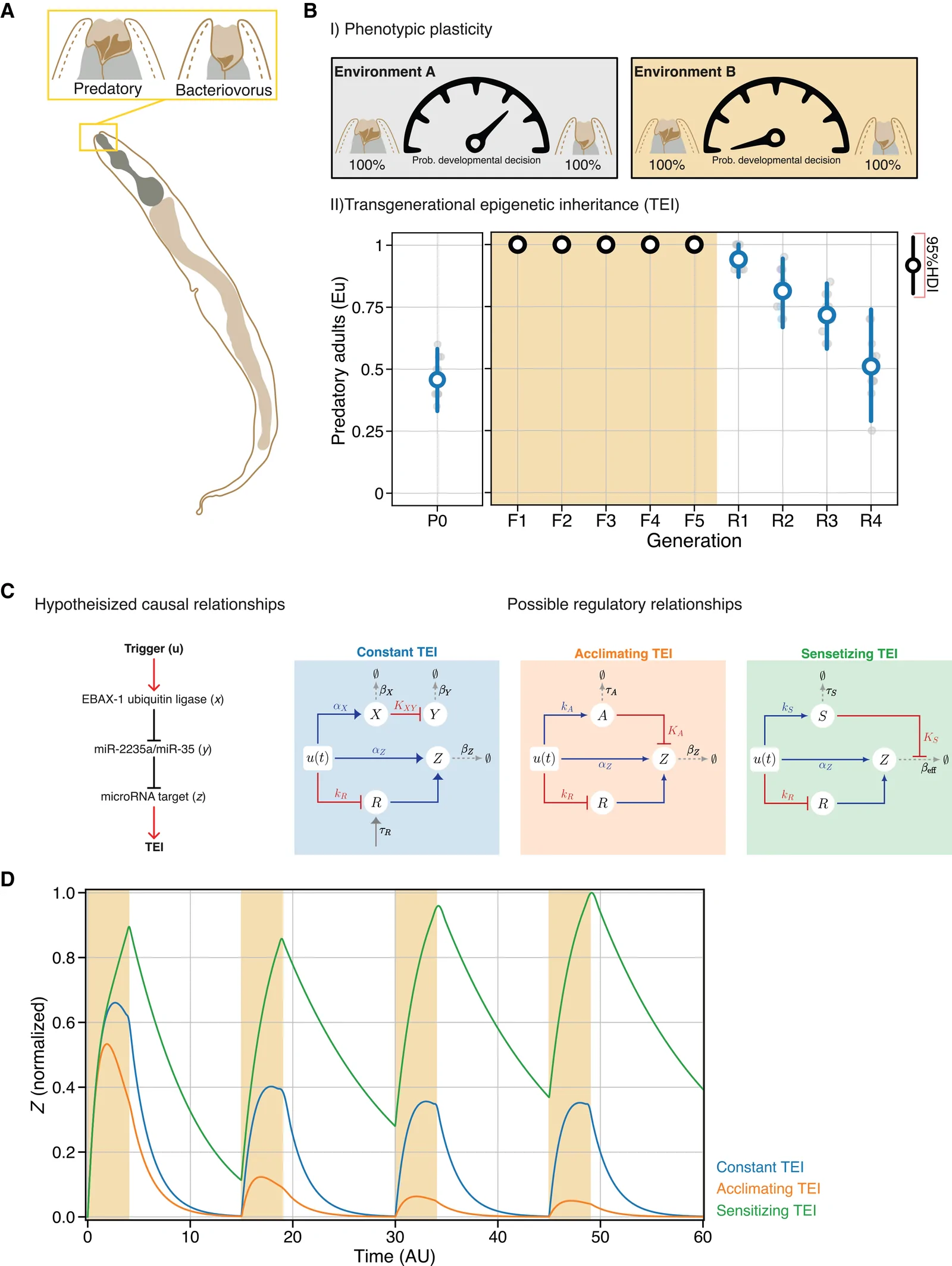
Mouth-form polyphenism in *P. pacificus* exhibits transgenerational epigenetic inheritance whose dynamics could follow constant, acclimating, or sensitizing theoretical models (A) The nematode *P. pacificus* develops either of two mouth forms: a wide eurystomatous (Eu) form which enables predation of other nematodes in addition to bacterivory, and a narrow non-predatory stenostomatous (St) form. The irreversible decision to express a mouth form is taken during the development from juvenile to adult stage. (B) The mouth-form polyphenism in *P. pacificus* is characterized by change in the probability of the developmental switch between the predatory and non-predatory mouth forms depending on the environmental conditions (I), and transgenerational epigenetic inheritance (TEI), where an exposure for at least five generations to an inducing environment results in epigenetic inheritance for three generations in the non-inducing environment (II). P0 indicates the probability of developing adults with the predatory mouth form in the naive worms. Vertical lines indicate the 95% highest density interval (HDI) for the predictive probability for the development of the predatory mouth $\tilde{\theta }$, inferred using a Bayesian model fitted to observed number of predatory adults out of 20 adults assayed per plate (see STAR Methods). (C) We have previously suggested a hypothetical causal model which connect the environmental trigger to TEI.^14^ However, different regulatory models can be constructed based on our experimentally derived causal model. A constant TEI model describes a case where TEI is independent of environmental variability. An acclimating TEI describes a scenario in which repeated exposure to the environmental trigger dampens the TEI. Finally, in a sensitizing TEI model, the TEI amplifies with more periodic exposure to the inducing environment. Each model of TEI reflects a distinct regulatory network. (D) The dynamics of the three models of TEI with periodic exposure to the environmental trigger. The parameters used for the models can be found in Table S1. AU stands for arbitrary unit. The shaded areas in part II of (B and D) indicate the exposure to an environment that induces the development of the predatory mouth form in *P. pacificus*.

The nematode *Pristionchus pacificus* provides a perfect model to study TEI.^12^ This worm exhibits developmental plasticity of its mouth parts with two alternative forms in response to environmental variation.^13^ Specifically, *P. pacificus* individuals adopt a *stenostomatous* (St) morph with a single tooth and a narrow buccal cavity, or alternatively, a *eurystomatous* (Eu) morph with two teeth and a broad cavity (Figure 1A). This developmental decision is irreversible and affects the feeding capabilities of adult worms. St individuals are strict bacterial feeders, whereas Eu animals are omnivores that can also kill other nematodes, including cannibalizing of conspecific worms, which is modulated via a complex self-recognition system.^15^ While *P. pacificus* adults do not kill kin, they will attack and consume individuals of other genotypes.^16^ As *P. pacificus* is a self-fertilizing hermaphrodite with many of the same appealing characteristics as *C. elegans* (short-generation time, easy husbandry and isogenic propagation), the regulatory mechanisms controlling mouth-form plasticity have been investigated by forward and reverse genetics. By now, a detailed gene regulatory network has been identified.^17^^,^^18^^,^^19^^,^^20^^,^^21^ Importantly, the mouth-form decision in *P. pacificus* is in parts stochastic in nature and is deeply influenced by a slew of environmental factors, most notably diet.^22^^,^^23^^,^^24^ Together, this mouth form polyphenism results in intraguild life-history predation that could greatly shape the ecological dynamics of communities of *P. pacificus* and its relatives in the wild.^16^^,^^25^

In nature, *P. pacificus* is found in association with beetles of the family Scarabaeidae.^26^ Specifically, dauer larvae of *P. pacificus* and other nematodes reside on adult beetles (for review see Sommer^27^). Following the natural death of a beetle in the soil, dauer larvae develop into adults, forming a short-lived community sustained by bacteria and other microbes that decompose the insect carcass. Subsequently, new dauer larvae disperse into the environment in search of new beetle hosts. In the context of the beetle life cycle, mouth-form polyphenism in *P. pacificus* with its associated predation is expected to greatly affect the dynamics of the transient ecological competition that unfolds on the decaying carcass. Specifically, previous studies have shown that transition from juvenile to adult through the dauer stage on a beetle carcass induces predatory moth-form,^28^ and, fascinatingly, the likelihood of developing into dauer larvae in one *P. pacificus* isolate can be greatly influenced by pheromones secreted from competing isolates.^29^ However, despite multiple recent studies on the ecological significance of phenotypic plasticity in *P. pacificus* in promoting and/or hindering co-existence,^23^^,^^24^^,^^30^ little is known about the role of epigenetic processes and potential TEI of the predatory mouth form in the wild, particularly in the context of the decaying beetle carcasses.

Recently, we demonstrated that dietary induction of the predatory mouth form can be transgenerationally inherited for multiple generations.^14^ Using a wild isolate of *P. pacificus* (RSC011) from a high altitude location on La Réunion Island, a long-term environmental induction experiment has been performed. When grown on the standard laboratory food source *Escherichia coli* OP50, a *P. pacificus* PSC011 adult has around 30% chance of developing the Eu mouth form.^23^ In contrast, exposure of these worms to *Novosphingobium* L76—a bacterium derived from *Pristionchus*’s natural environment on La Réunion^22^—increases the probability of developing the Eu mouth form to 100%^14^ (Figure 1B). Specifically, this dietary induction of the predatory mouth form is immediate (in the first generation of exposure), systemic (found in all the lines exposed to the inducing diet), and complete (all the progeny of an exposed mother show the Eu morph). Strikingly, dietary reversal from *Novosphingobium* L76 to *E. coli* OP50 will result in the TEI of the predatory mouth form^14^ (Figure 1B). This transgenerational memory was observed for a minimum of four generations, but required an original exposure to *Novosphingobium* L76 for at least five generations. This pattern of TEI is phenomenologically different from the majority of examples of TEI observed in *C. elegans*.^14^ Subsequently, we have shown that bacterial-derived vitamin B12—through its role in bringing about vitellogenin provisioning—is necessary and sufficient to induce TEI of predatory mouth form in *P. pacificus*.^31^

It is important to note that these laboratory studies do not mimic the conditions in nature. In the ecological context of decaying scarab beetles, spatial and temporal variability with respect to diversity of bacteria in the growing microbial biomass on the carcass is expected. To explore how such variability in the exposure to the inducing agent might affect the TEI of the predatory morph in *P. pacificus*, we designed multiple alternative induction experiments in which *P. pacificus* is alternatively exposed to *Novosphingobium* L76 or *E. coli* OP50 for different numbers of generations.

In this manuscript, firstly, we develop simple theoretical models that indicate the potential patterns of TEI in *P. pacificus* as a function of environmental variability. Secondly, we introduce a two-pronged experimental approach to understand which theoretical model better explains the TEI of mouth-form polyphenism. Our approach relies on two experimental designs: (a) alternative induction experiment, which enables a systematic exploration of how TEI behaves as a function of environmental variability and (b) superinduction experiment, designed to assess the behavior of TEI with respect to the intensity of the environmental cue. Taken together, our results shed light on the dynamics of TEI as a function of environmental variability, and they link the observed changes to a theoretical framework, thus adding to a growing body of mechanistic understanding of the TEI of the predatory morph in *P. pacificus*.

## Results

### The expected dynamics of TEI

There have been several theoretical attempts to incorporate epigenetic inheritance in an evolutionary framework.^8^^,^^32^ However, such models are primarily concerned with the potential role of epigenetic inheritance as an additional source of correlation between parents and offspring and its consequence on the response of the population to selection. In contrast, we are interested in the dynamics of TEI itself, as a function of the environment. A suitable model in this context was previously introduced by Houri-Zeevi et al.^11^:

(Equation 1) $$\frac{dz}{dt}=xy+\gamma \frac{z^{n}}{z^{n}+k^{n}}-\alpha z,$$

where z is the state of a gene which has been silenced by trigger x, which is present only at $t_{0}$ and decays with rate α. This model is based on three rules observed with respect to the TEI of gene silencing induced by double-stranded RNA in *C. elegans*: (1) correlation between offspring response and parental state, (2) stochastic inheritance state (y) in parents that are exposed to the trigger, and (3) transgenerational momentum, i.e., the likelihood of TEI increases the longer it persists.

The TEI of mouth-form state in *P. pacificus* seems to deviate from rules (2) and (3): the response to the environmental trigger (*Novosphingobium* L76) was systemic, resulting in all the lines assuming predatory mouth form, and little variation was observed in TEI amongst lines that were exposed to trigger from 5 to 95 generations.^14^ For an individual i of strain j, its mouth-form state $X_{i}$, in environment $E_{1}$ can be described as $X_{i}∼Binom\left(P_{j|E_{1}}\right)$, and when exposed to $E_{2}$, $X_{i}∼Binom\left(P_{j|E_{2}}\right)$, where $P_{j}$ is the probability of developing either of the two mouth forms in a given environment. However, TEI implies $P_{j|E_{2}}$ should be affected by the environment experienced by the parent. We propose a model of TEI in *P. pacificus*,^14^ represented by a causal network:

(Equation 2) Tigger→X⊣Y⊣Z,

where an unknown trigger induces the activity of enzyme X (EBAX-1 ubiquitin ligase), which suppresses the microRNA Y (miR-2235a/miR-35). In the absence of the trigger or following the degradation of enzyme X, Y degrades a microRNA target (Z), which prevents the TEI of the predatory mouth-form in *P. pacificus*. Although more mechanistic details of this process—at least for the moment—remain elusive, this causal model can enable us to construct a phenomenological model. A simple circuit could be specified as

(Equation 3) $$\begin{array}{cc} \frac{dX}{dt} & =\alpha_{X}u\left(t\right)-\beta_{X}X\\ \frac{dY}{dt} & =\frac{\alpha_{Y}}{1+{\left(\frac{X}{K_{XY}}\right)}^{n_{XY}}}-\beta_{Y}Y\\ \frac{dR}{dt} & =\frac{1-R}{\tau_{R}}-k_{R}u\left(t\right)R, \end{array}$$

where u(t) is the trigger at time t, α indicates maximum production, and β reflects degradation. An additional component R is added, simply to enable an explicit inclusion of history in the system. The dynamics of Z can be modeled to match different forms of TEI. A constant TEI can be achieved by assuming a direct relationship between Z and the effect of the trigger u and the available R:

(Equation 4) $$\frac{dZ}{dt}=\alpha_{Z}u\left(t\right)R-\beta_{Z}Z.$$

An incoherent feedforward loop can be used to model an acclimating TEI response, which exhibits a weaker TEI pattern given a history of intermittent exposure to the trigger:

(Equation 5) $$\begin{array}{cc} \frac{dA}{dt} & =k_{A}u\left(t\right)-\frac{A}{\tau_{A}}\\ \frac{dZ}{dt} & =\frac{\alpha_{Z}u\left(t\right)R}{1+\gamma_{A}A}-\beta_{Z}Z, \end{array}$$

where A acts as an inhibitor that builds up during the induction phase. Finally, a sensitizing TEI, which exhibits stronger patterns of TEI if it was exposed to the trigger in the past, can be modeled by including S to protect Z from degradation:

(Equation 6) $$\begin{array}{cc} \frac{dS}{dt} & =k_{S}u\left(t\right)-\frac{S}{\tau_{S}}\\ \beta_{\text{eff}} & =\frac{\beta_{Z}}{1+c_{S}S}\\ \frac{dZ}{dt} & =\alpha_{Z}u\left(t\right)R-\beta_{\text{eff}}Z. \end{array}$$

Each model results in a distinct regulatory network (Figure 1C), each with a distinct response to environmental change: A constant TEI model describes a case where TEI is independent of environmental variability. An acclimating TEI describes a scenario in which repeated exposure to the environmental trigger dampens the TEI. Finally, in a sensitizing TEI model, the TEI amplifies with more periodic exposure to the inducing environment.

These models illustrate three general patterns of TEI that one could observe in *P. pacificus* (Figure 1D). Neither of the three models can *a priori* be ruled out, and all could be justified in an ecological and/or evolutionary context. Although the details of these models are not a direct reflection of data, they provide us with toy models to guide our experimental design.

### A black-box approach to relate theory to reality

To relate these theoretical expectations to reality, we designed two types of experimental approaches: an alternative induction experiment and a superinduction experiment. The reasoning behind these designs is to treat the gene regulatory circuitry responsible for the induction of the predatory Eu mouth form in *P. pacificus* as a black box^33^: since the inner workings of the molecular machinery are not fully understood, by manipulating the input (environmental cue) and observing the output (the pattern of TEI) we aim to reveal the dynamics of this system. Specifically, given the ecological ramifications of the development of the predatory mouth form in *P. pacificus*, treating the machinery behind the TEI as a black box enables us to infer the relationship between environmental variability—including temporal patterns of variability—and the pattern of TEI, thus providing a more accurate starting point to understand the ecological and evolutionary consequences of the non-genetic inheritance of a cannibalistic trait.

### The design of the alternative induction experiment

**Figure 2:**
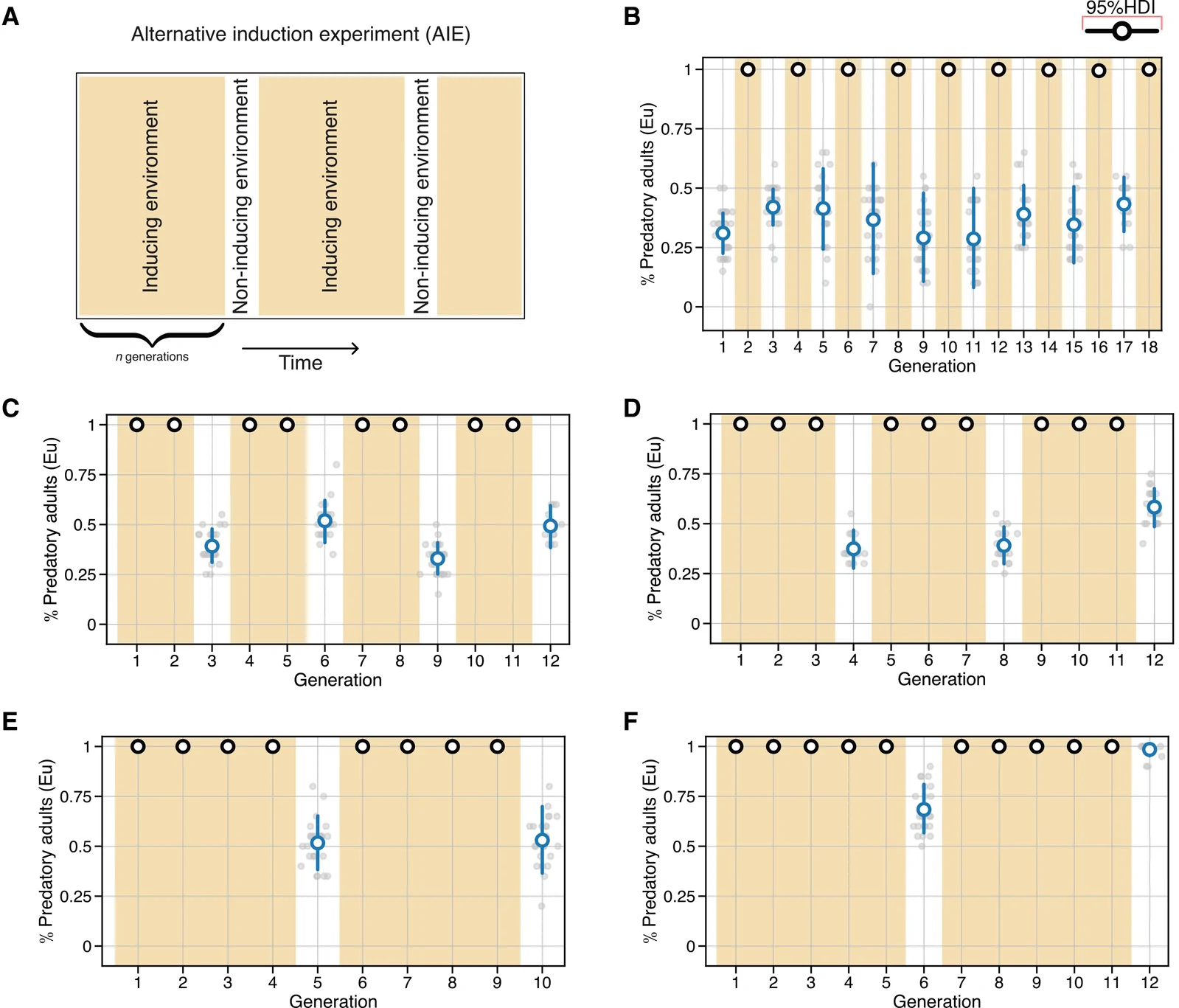
Alternative induction experiments reveal that TEI of the predatory mouth form is largely history-independent, except after five generations of exposure, which produces a sensitizing response (A) The design of the alternative induction experiment (AIE). (B–F) AIE with n=1 result in no accumulating of TEI over the course of the experiment (B), suggesting that in this scenario the TEI does not sensitize and instead resets by the removal of the environmental trigger. In AIE with n=2 (C), n=3 (D), and n=4 (E), the same constant response in TEI, independent of the duration of exposure to the environmental trigger, is observed. However, with n=5 (F), a sensitizing dynamics is observed, as the TEI is elevated after the second phase of exposure. In (B)–(F), vertical lines indicate the 95% highest density interval (HDI) for the predictive probability for the development of the predatory mouth $\tilde{\theta }$. The shaded areas in each figure indicate propagation on the inducing diet *Novosphingobium* L76.

At the start of the experiment, a single self-fertilizing hermaphrodite of *P. pacificus* RSC011 is transferred from the standard *E. coli* OP50 (non-inducing environment) to *Novosphingobium* L76 (inducing environment). In each generation, a single healthy J4 juvenile progeny was moved to a new plate. The single-worm bottleneck means that the population genetic changes are driven by drift; following the same procedure as described in Quiobe et al.^14^ After propagation on *Novosphingobium* L76 for n generations, the next generation would be propagated on *E. coli* OP50, with the following generation being transferred back to *Novosphingobium* L76. This pattern is repeated during the experiment. The alternative exposure to *Novosphingobium* L76 determines the number of generations during which the worms are exposed to the trigger, before the interruption of the exposure for a single generation (Figure 2A). We use alternative induction experiments (AIEs) with n=1,2,3,4,5 to illustrate the dynamics of TEI in *P. pacificus* when exposed to changing environments.

### The design of the superinduction experiment

In contrast to the AIE, in which the duration and the repetition of the induction are manipulated, in a superinduction experiment, we manipulate the intensity of the environmental cue. Previously, we have shown that vitamin B12 is both necessary and sufficient to induce TEI of the predatory mouth form in *P. pacificus*.^31^ To increase the intensity of the inducing agent, we supplement *Novosphingobium* L76 with 1500nM vitamin B12. In a superinduction experiment, nematodes are propagated on the supplemented diet for n generations, where a single healthy J4 juvenile progeny was moved to a new plate to start a new generation. Following n generations of superinduction, nematodes are propagated on *E. coli* OP50 plates, to observe the pattern of TEI of the predatory mouth form. To infer the effect of superinduction, each experiment is complemented by an induction experiment, in which nematodes are propagated on *Novosphingobium* L76 for n generations before being transferred to *E. coli* OP50. We hypothesized that the manipulation of the concentration of trigger in the superinduction experiment would supplement the AIE design to reveal the dynamics of TEI in *P. pacificus*.

### The TEI of cannibalism in *P. pacificus* follows a mixed model

In the AIE, nematodes are grown for n generations to the inducing environment (*Novosphingobium* L76), followed by a single generation of growth on the non-inducing environment (*Escherichia coli* OP50). This pattern is repeated during the experiment to infer the dynamics of the TEI of mouth-form state as a function of environmental variability. A single hermaphrodite is used to establish a new population in each generation, ensuring that the observed patterns are not due to selection. In AIE with n=1, i.e., a single generation of exposure, a sensitizing TEI would result in an increasing probability of developing the predatory mouth form in the non-inducing environment. However, in such an AIE, no clear trend is observed in 18 generations (Figure 2B). This lack of pattern becomes evident when comparing the predictive risk difference (RD) for the probability of developing the predatory mouth form between generations grown on the non-inducing diet (Figure S3). Similarly, no such trend was observed in n=2 (Figures 2C and S4A). These results hint at a history-independent TEI that only switches on following a minimum of 5 generations of exposure to the inducing environment, as previously observed in Quiobe et al.^14^ The AIE with n=3 and n=4, both were consistent with this model (Figures 2D and 2E). However, for n=3, comparison between generation 4 and 12, 0.076≤ 95%HDI(RD)≤0.34, suggesting an effect on the response on non-inducing diet (Figure S4B). In contrast, AIE with n=5—which corresponds to the minimum number of generations required for TEI of predatory mouth form in *P. pacificus*—exhibited a pattern consistent with a sensitizing TEI: a second exposure to the inducing environment for five generations increased the probability of assuming the predatory mouth form on the non-inducing environment (0.17≤ 95%HDI(RD)≤0.43 when comparing generation 12 with 6) (Figures 2F and S4D) (RD for all experiments in 2B–2F are found in Figures S3 and S4).

### Trigger concentration modulates TEI of cannibalism in *P. pacificus*

We have previously proposed that the key trigger of the TEI of mouth-form polyphenism in *P. pacificus* is vitamin B12, which is produced by *Novosphingobium* L76.^31^ Consequently, provided that the uptake of the trigger by the worm is not the limiting factor, supplementing the inducing diet (*Novosphingobium* L76) with additional B12 would increase the concentration of the trigger that the worm encounters. Increase in the concentration of the trigger can result in the early onset of TEI and/or change the duration of TEI. To test our hypothesis, we designed the superinduction experiment.

**Figure 3:**
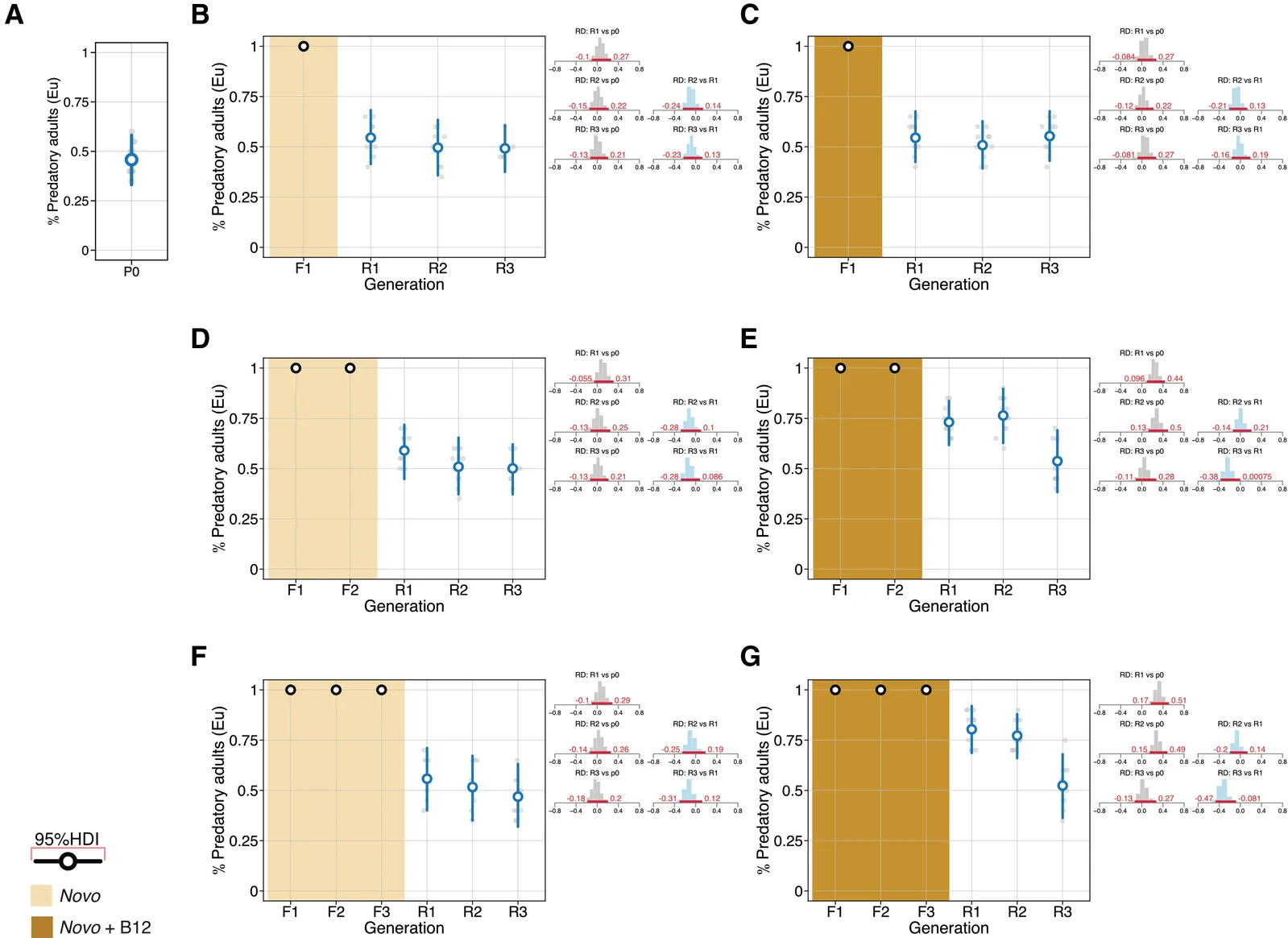
Superinduction with vitamin B12 accelerates the onset of the epigenetic inheritance of the predatory mouth form relative to induction alone (A–G) The pattern of TEI in induction (B, D, and F) and superinduction experiments (C, E, and G). In each experiment, worms were propagated on *Novosphingobium* L76 (induction) or *Novosphingobium* L76 plus 1500nM vitamin B12 (superinduction) for n∈{1,2,3} generations before being transferred back to the non-inducing environment (*E. coli* OP50). The probability of developing the predatory mouth from in the naive worm in *E. coli* OP50 is shown as our reference (A). Vertical lines indicate the 95% HDI for the predictive probability for the development of the predatory mouth $\tilde{\theta }$. To illustrate the pattern of TEI in each experiment, we measured RD based on the posterior predictive for $\tilde{\theta }$ during reversal to the non-inducing environment following induction or superinduction related to that of the P0 worm (see STAR Methods). This analysis quantifies the convergence toward the expected response. Additionally, we measured the RD between generation m in the non-inducing environment (Rm) relative to that of the first generation grown on the non-inducing environment following induction or superinduction (R1). This comparison indicates the pattern epigenetic inheritance. The posterior predictive for RD is shown as histograms, with the horizontal red bar indicating the 95% HDI for RD for a given comparison.

In the superinduction experiment, we exposed *P. pacificus* RSC011 worms to either *Novosphingobium* L76 (inducing condition) or *Novosphingobium* L76 plus 1500nM vitamin B12 (superinducing condition) and then transferred them back to the non-inducing environment (*E. coli* OP50). In the absence of any information in regard to the effect of B12 concentration on TEI, we explored a range of induction and superinduction periods ranging from one to five generations (Figure 3). We have previously reported that a minimum of five generations of exposure to *Novosphingobium* L76 is required to induce TEI in *P. pacificus* RSC011.^14^

A single generation of exposure under inducing or superinducing conditions has no credible effect on the predictive probability of developing the predatory mouth form in the non-inducing environment (Figures 3B and 3C). Exposure for two generations to the superinducing condition elevates the predictive probability of developing the predatory mouth form in the first generation in the non-inducing environment (For inducing, $0.45\leq 95\%HDI\left({\tilde{\theta }}_{\text{R}1}\right)\leq 0.72$; for superinducing, $0.62\leq 95\%HDI\left({\tilde{\theta }}_{\text{R}1}\right)\leq 0.84$) (Figures 3D and 3E). The RD for the superinducing condition suggests an effect when comparing the first generation on non-inducing diet (R1) and the third generation (R3) (−0.38≤ 95%$HDI\left({RD}_{\text{R}3\;\;\text{vs}\;\;\text{R}1}\right)\leq 0.0$).

The effect of superinduction in early onset of TEI becomes evident when comparing experiments in which induction or superinduction was carried out for three generations (Figures 3F and 3G). Whereas ${\tilde{\theta }}_{\text{R}i}$ shows a minor effect following induction, in the superinduction case, $0.69\leq 95\%HDI\left({\tilde{\theta }}_{\text{R}1}\right)\leq 0.92$, which remains high for the second generation in the non-inducing environment ($0.66\leq 95\%HDI\left({\tilde{\theta }}_{\text{R}2}\right)\leq 0.88$), before going to levels comparable with the inducing case.

**Figure 4:**
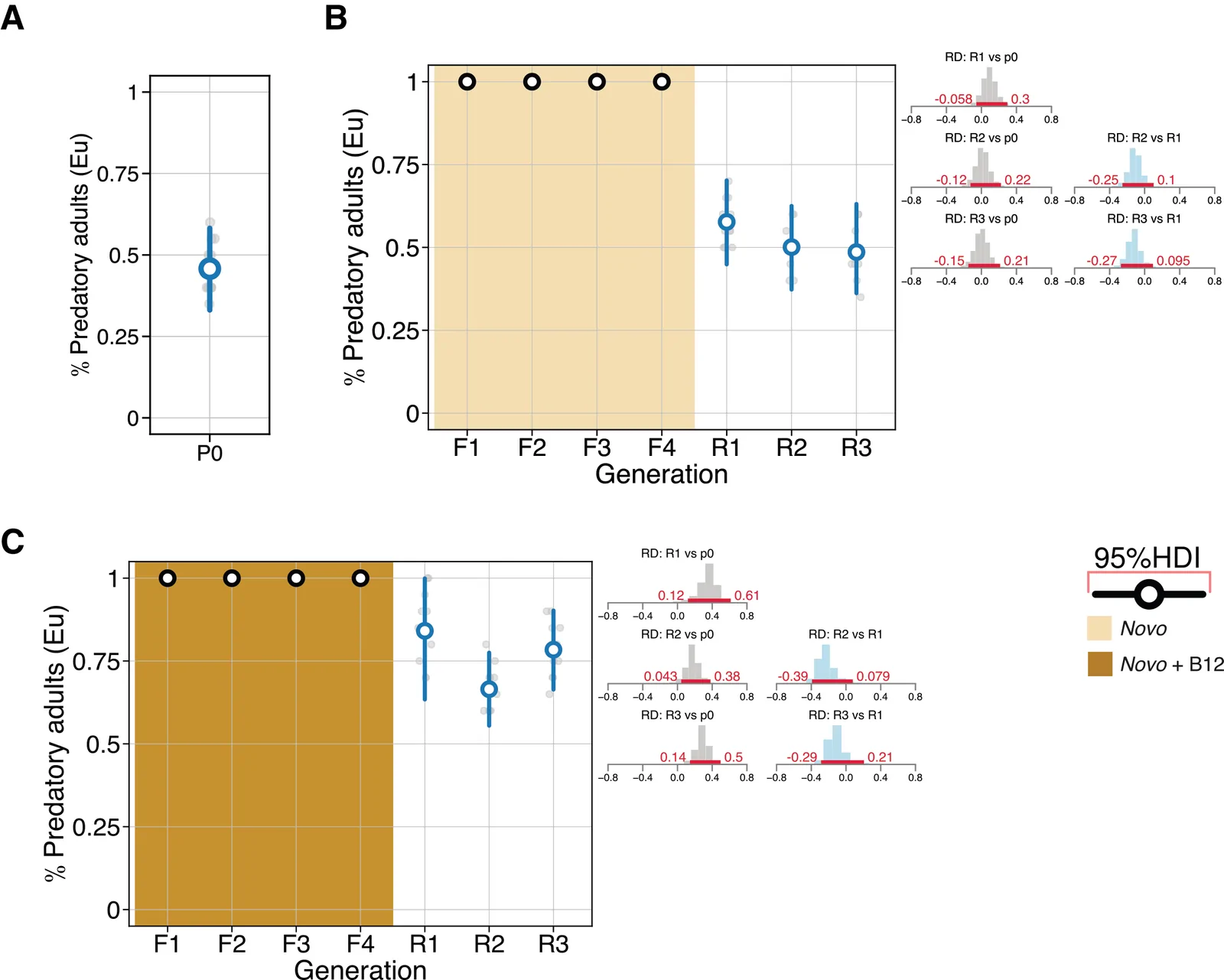
Four generations of induction or superinduction reveal distinct patterns of epigenetic inheritance (A–C) The pattern of TEI following four generations of induction, i.e., propagation on *Novosphingobium* L76 (B), and superinduction, i.e., propagation on *Novosphingobium* L76 plus 1500nM vitamin B12 (C). The probability of developing the predatory mouth from in the naive worm in *E. coli* OP50 is shown as our reference (A). Vertical lines indicate the 95% HDI for the predictive probability for the development of the predatory mouth $\tilde{\theta }$. We measured RD between $\tilde{\theta }$ of a given generation during reversal and P0, as an indication of convergence to the naive response, and RD of $\tilde{\theta }$ during reversal, as an illustration of epigenetic inheritance. Each horizontal red bar indicates the 95% HDI for RD for a given comparison.

Induction and superinduction for four generations results in a striking pattern: the probability of developing the predatory mouth form on the non-inducing diet immediately after superinduction is very high $\left(0.63\leq 95\%HDI\left({\tilde{\theta }}_{\text{R}1}\right)\leq 1\right)$ compared with the inducing case $\left(0.45\leq 95\%HDI\left({\tilde{\theta }}_{\text{R}1}\right)\leq 0.7\right)$ (Figures 4B and 4C). However, the second generation in the non-inducing environment following superinduction shows a decline in the probability of developing the predatory mouth form ($0.55\leq 95\%HDI\left({\tilde{\theta }}_{\text{R}2}\right)\leq 0.77$), which is followed by an increase in the following generation $\left(0.66\leq 95\%HDI\left({\tilde{\theta }}_{\text{R}3}\right)\leq 0.9\right)$. It remains to be seen to what extent such effects on TEI can be attributed to the broader effects of vitamin B12 on the nematode.

**Figure 5:**
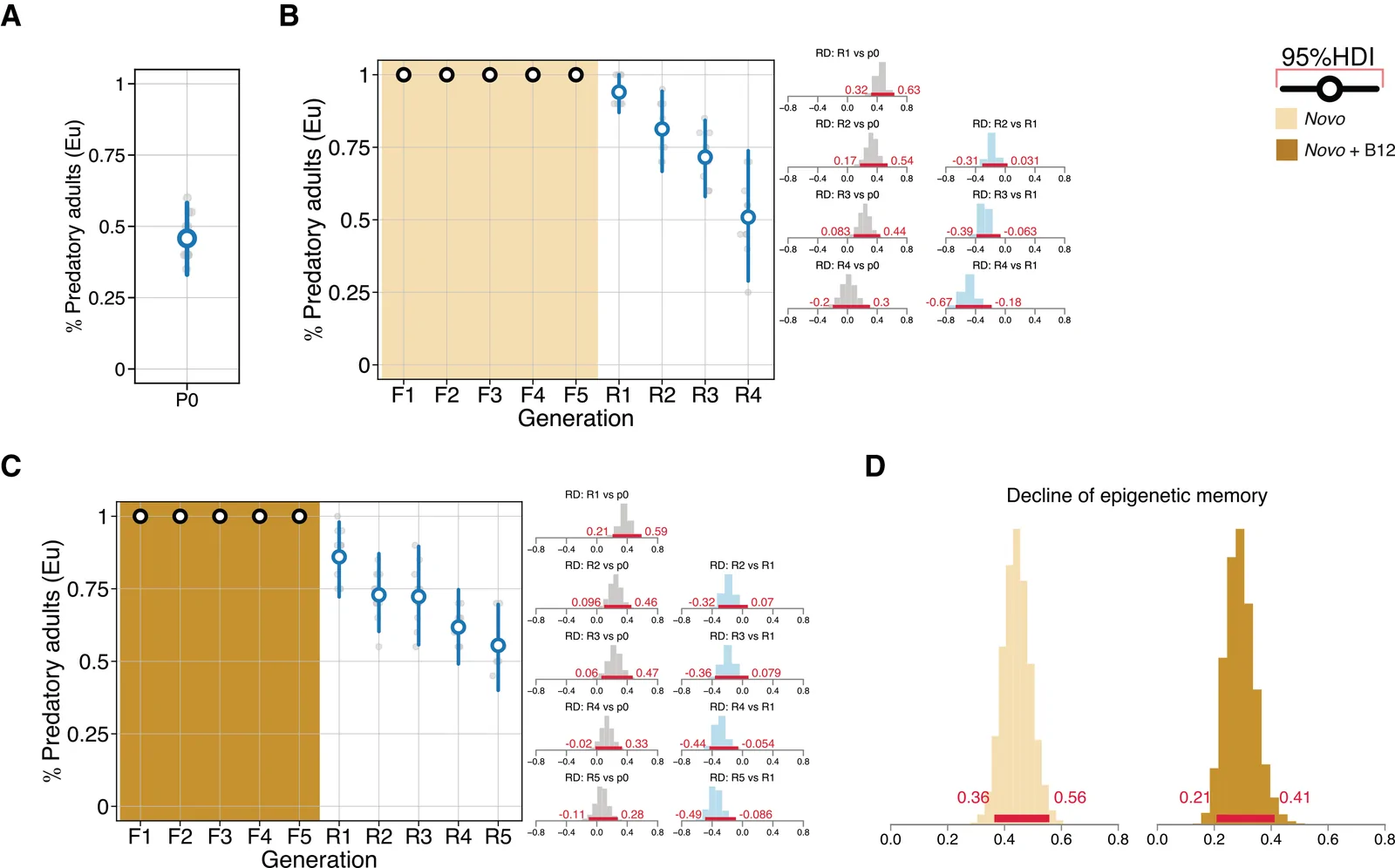
Five generations of superinduction slow the decay of transgenerational epigenetic memory of the predatory mouth form compared with induction alone (A–D) The pattern of TEI following five generations of induction, i.e., propagation on *Novosphingobium* L76 (B), and superinduction, i.e., propagation on *Novosphingobium* L76 plus 1500nM vitamin B12 (C). The probability of developing the predatory mouth from in the naive worm in *E. coli* OP50 is shown as our reference (A). Vertical lines indicate the 95% HDI for the predictive probability for the development of the predatory mouth $\tilde{\theta }$. We measured RD between $\tilde{\theta }$ of a given generation during reversal and P0, as an indication of convergence to the naive response, and RD of $\tilde{\theta }$ during reversal, as an illustration of epigenetic inheritance. In (B) and (C), each horizontal red bar indicates the 95% HDI for RD for a given comparison (D) The rate of decline in epigenetic inheritance between (B) and (C) was estimated using a Bayesian model (STAR Methods: Equations 10, 11, and 12). The histograms represent the posterior predictive distribution for the rate of decline in epigenetic memory (κ, STAR Methods: Equation 12), with horizontal red bars indicating the 95% HDI for κ.

Induction and superinduction for five generations clearly demonstrate the effect of the concentration of the trigger molecule on the duration of the TEI of mouth-form polyphenism (Figures 5B and 5C). Consistent with our previous findings,^14^ five generations of exposure to the inducing conditions result in an elevated probability of developing the predatory mouth form immediately following induction ($0.87\leq 95\%HDI\left({\tilde{\theta }}_{\text{R}1}\right)\leq 0.1$). This response gradually declines, finally reaching $0.29\leq 95\%HDI\left({\tilde{\theta }}_{\text{R}4}\right)\leq 0.74$ in our experiment (Figures 4B and S5C). However, the superinduction slows the rate of decline of the TEI, resulting in $0.49\leq 95\%HDI\left({\tilde{\theta }}_{\text{R}4}\right)\leq 0.75$ after four generations on the non-inducing environment and remains relatively high in the subsequent generation $\left(0.4\leq 95\%HDI\left({\tilde{\theta }}_{\text{R}5}\right)\leq 0.7\right)$(Figure 5C). Given that for the n=5 superinduction experiment, the RD between R4 and P0 remained high (−0.02≤ 95%$HDI\left({RD}_{\text{R}4\;\;\text{vs}\;\;\text{P0}}\right)\leq 0.33$), whereas for the induction experiment the corresponding RD more-or-less centered around zero (−0.2≤ 95%$HDI\left({RD}_{\text{R}4\;\;\text{vs}\;\;\text{P0}}\right)\leq 0.3$), we continued the superinduction experiment for an additional generation. Interestingly, the RD between R5 and P0 still remained higher than that of the induction experiment at R4 (−0.11≤ 95%$HDI\left({RD}_{\text{R}5\;\;\text{vs}\;\;\text{P0}}\right)\leq 0.28$). The difference between the decline in epigenetic memory between induction and superinduction for n=5 can be better illustrated using our exponential decay model (Equations 10, 11, and 12). Our model indicates that in the induction experiment, the epigenetic memory declines faster than in the superinduction experiment (0.36≤ 95%HDI(κ)≤0.56 for the induction experiment vs. 0.21≤ 95%HDI(κ)≤0.41 for the superinduction) (Figure 5D).

## Discussion

In this study, we investigated the logic and dynamics of TEI of a predatory trait. The nematode *P. pacificus* is well-suited to investigate TEI in response to environmental variability because it exhibits one of a few cases of TEI with an ecologically-relevant phenotype. Building on simple theoretical models indicating the potential patterns of TEI of the predatory mouth form in *P. pacificus*, we performed two sets of experiments. Alternative induction experiments revealed that TEI of the predatory morph is largely history-independent. Superinduction experiments, on the other hand, indicated that an earlier onset of TEI is possible, suggesting that the nematode itself is not the limiting factor of the initial TEI. These findings help to further increase our understanding of this complex memory phenotype and add information necessary to better understand the relevance of this phenomenon in nature.

Alternative induction experiments followed a mixed model with AIE with n<5 indicated as history-independent, whereas AIE with *n* = 5 exhibited a sensitized TEI (Figure 2). The pattern in AIE with n=5 is puzzling: why didn’t the interruption in this AIE reset the effect in non-inducing environment, while it was enough in AIEs with n<5 to disrupt the memory? One possible strategy to resolve this puzzle is to conduct AIEs on a longer temporal scale to see if enough triggers accumulate over longer periods. However, that solution complicates the issue since random mutations can fix in AIEs, given its similarity to a mutation-accumulation experiment. Previously,^14^ we documented such mutations during the long-term experimental induction. We reasoned that the pattern observed in AIEs, with clear sensitization in AIE with n=5, can be explained by two alternative hypotheses: (1) there is a limit to the amount of trigger absorbed by a worm per generation and five generations are needed to accumulate enough trigger across generations to switch on the TEI or (2) the concentration of the trigger in *Novosphingobium* L76 is not enough, and if presented with higher amount of trigger, *P. pacificus* would express TEI after shorter exposure to the inducing environment.

Our superinduction experiments strongly favor the second model as vitamin B12 supplementation on top of a *Novosphingobium* diet will result in higher transmission of the predatory mouth form (Figure 3). It has to be noted that the exact concentrations of vitamin B12 in individual worms cannot be determined, as reliable vitamin B12 sensors are still not available.^34^^,^^35^ While the concentrations of vitamin B12 in these supplementation experiments followed those of previous studies on surplus killing^22^ and transgenerational memory,^14^ *Pristionchus* vitamin B12 applications have to use higher doses than those used in *C. elegans* (1,000 or 1,500 nM versus 63 nM). Interestingly, previous studies showed that the induction of the predatory mouth form requires much less vitamin B12, i.e., supplementation of around 1nM is sufficient, whereas only very high, in parts, supraphysiological concentrations can cause the TEI after vitamin B12 supplementation. Despite the difficulty in measuring B12 concentration in worms, the effect of exogenous supplementation can be observed at the transcriptional level. For example, we have recently shown how the effects of B12 and *Novosphingobium* L76 on transcription, including upregulation in 8 out of the 9 identified vitellogenin genes in *P. pacificus*.^31^

The observation that the TEI of predatory mouth form in *P. pacificus* does not diminish as a function of periodic exposure to an inducing environment (acclimating TEI), but rather follows a mixture of constant and sensitizing TEI models, with a strong role for the concentration of the inducing agent, hints at just how the inclusion of the TEI infuses the ecological competition between *P. pacificus* isolates and other nematode species with more complexity. Specifically, it remains to be seen how repeated superinduction would affect the dynamics of TEI, and, more fundamentally, if superinduction has its counterparts in the natural habitat of *P. pacificus*. More importantly, the decaying beetle—where *P. pacificus* is reliably found—is decimated by a bacterial community. Understanding how a bacterial community would affect the TEI of predatory mouth form in *P. pacificus* is crucial to grasp the ecological consequences of TEI in *P. pacificus* and the extent to which the dynamics of the TEI itself could be subject to selection.

Transgenerational plasticity (TGP) is a broad term that describes non-genetic inheritance when the effect of the environment in one generation affects a subsequent generation.^36^ Such a broad concept is adequate when discussing our results, especially the results of the superinduction experiments, where TEI, with its strict criterion concerning the number of affected generations, does not reflect the complexity of the observed dynamics. Additionally, our results illustrate that the insights gained from our experimental designs reveal the intricacies beneath these terms once one delves into the detailed behavior of an exemplar of TGP. Specifically, our results indicate how the induction of developmental plasticity in *P. pacificus* and the patterns of TGP are not one and the same. Rather, while the induction of the predatory mouth form is systemic and immediate, the onset of TGP is both influenced by the number of generations during which the worms are exposed to the environmental trigger and the concentration of that trigger, best described as a combination of the constant and sensitizing TEI models (Figure 1D).

It is assumed that the evolution of TEI is directly dependent on environmental variability, where the evolution of developmental plasticity itself is possible when within-generation environmental variability is low and between-generation variability exists and TEI requires longer periods of stability.^37^ Our exploration of TEI of a cannibalistic trait in *P. pacificus* provides a unique example to understand how epigenetic inheritance unfolds in a trait that directly affects competition via predation in an organism that lives in a complex yet tractable ecological setting. Luther Burbanks’ use of “ever-moving life forces” in his description of the role of environment on heredity^38^ is an apt description of the attempt to better understand the dynamics of non-genetic inheritance, a necessity if we are to characterize the role of non-genetic inheritance in ecology and evolution.

### Limitations of the study

(1) Other regulatory relationships can be envisioned. Further experimental studies on the regulatory network underpinning the TEI of the mouth-form state in *P. pacificus* will likely require revising the currently hypothesized regulatory relationships. (2) The durations of exposure to the inducing environment in AIEs were chosen rather arbitrarily. While they span a range that is experimentally tractable and sufficient to elicit TEI, they do not result from a systematic exploration of all possible exposure patterns. In principle, AIEs could be designed with variable durations of exposure to the inducing environment, such as incorporating irregular exposure intervals, asymmetric cycles, or stochastic sequences, to probe how different temporal patterns of induction shape the TEI response. However, not all such schemes for an AIE would be equally informative about the underlying regulatory relationships. (3) The effect of superinduction for more than five generations on TEI remains unexplored. Extending superinduction beyond the regimes tested here could, in principle, reveal whether the epigenetic memory of the predatory mouth form saturates, stabilizes, or begins to decay under prolonged exposure to elevated trigger concentrations. However, investigating longer superinduction regimes is further complicated by the potential unintended drawbacks of exposing *P. pacificus* worms to supraphysiological levels of B12 over many generations. These confounding effects would make it difficult to disentangle changes in TEI dynamics from broader toxicological or adaptive responses to long-term B12 supplementation, thereby limiting the interpretability of extended superinduction experiments.

## Resource availability

### Lead contact

Further information and requests for resources and reagents should be directed to and will be fulfilled by the lead contact, Ralf J. Sommer (ralf.sommer@tuebingen.mpg.de).

### Materials availability

This study did not generate new and/or unique reagents.

### Data and code availability

• Data reported in this paper are available on the GitHub repository associated with this manuscript (https://github.com/Kalirad/NematodeSuperinduction, https://doi.org/10.5281/zenodo.19183573).
• All code and software used to analyze the experimental data and the theoretical regulatory models are available on the GitHub repository associated with this manuscript.
• Any additional information required to reanalyze the data reported in this paper is available from the lead contact upon request.

## Acknowledgments

This work was funded by the 10.13039/501100004189Max Planck Society.

## Author contributions

S.P.Q., A.K., and R.J.S. designed the experiments. S.P.Q. and A.K. conducted the experiments. S.P.Q. collected the data. A.K. analyzed and visualized the results. A.K. did the mathematical modeling. S.P.Q., A.K., and R.J.S. wrote the manuscript.

## Declaration of interests

The authors declare no competing interests.

## STAR★Methods

### Key resources table

**Table:** 

REAGENT or RESOURCE	SOURCE	IDENTIFIER
**Experimental models: Organisms/strains**		

*P. pacificus* RSC011 strain	Sommer Lab-MPI Biology	RSC011

**Bacterial strains**		

*E. coli* OP50	N/A	N/A
*Novosphingobium* sp. L76	N/A	N/A
*Escherichia coli* Kanamycin resistant	N/A	N/A

**Chemicals**		

Vitamin B12, 98%+%9 (dry wt basis)	Thermo Scientific	Cat. No. A1489403 Lot: S281015

**Software and algorithms**		

PyMC	Abril-Pla et al.^39^	https://github.com/pymc-devs/pymc
NumPy	Harris et al.^40^	https://numpy.org/
SciPy	Virtanen et al.^41^	https://scipy.org/
Code	This paper	https://github.com/Kalirad/NematodeSuperinduction, https://doi.org/10.5281/zenodo.19183573

**Deposited data**		

Raw phenotyping data	This paper	https://github.com/Kalirad/NematodeSuperinduction, https://doi.org/10.5281/zenodo.19183573

### Experimental model and study participant details

#### Pristionchus pacificus RSC011

The ancestral *P. pacificus* RSC011 isolate was cryopreserved within the first 10 generations after its initial collection from Coteau Kerveguen, La Réunion. Worms were reared at 20°C under standard conditions on Nematode Growth Medium (NGM) plates seeded with either *E. coli* OP50 or *Novosphingobium* L76. Since *P. pacificus* is a self-fertilizing (androdioecious) hermaphroditic species, the sex-based analysis does not apply.

#### Bacterial food sources

Bacterial strains were cultured overnight in Lysogeny Broth (LB) with kanamycin (50μg/ml) when required. Specifically, OP50 was grown overnight at 37°C in LB without shaking while *Novosphingobium* L76 was grown at 30°C at 180 rpm. NGM plates were seeded with 300μl of overnight cultures and incubated for 2 days prior to use.

### Method details

#### Dietary induction and food reversals

Worms were propagated on *Novosphingobium* for 5 and 10 generations, respectively. To transfer nematodes from *Novosphingobium* L76 to *E. coli* OP50, worms were first moved to NGM plates supplemented with (50μg/ml) of kanamycin with a kanamycin resistant *E. coli* strain as food for one generation, before being reintroduced to standard *E. coli* OP50-seeded NGM plates for subsequent generations. The number of worms used in each experiment are reported in Table S2.

#### Mouth-form phenotyping

Mouth-from phenotyping was performed by observing the nematode buccal cavity using a Zeiss Discovery V.20 stereo microscope (× 150 magnification) based on mouth-form identities as described before.^17^ On each plate, 20 young-adult animals were assayed.

#### Dietary supplementation

Vitamin B12 (Cat. No. A1489403) – from Thermo Scientific – was dissolved in water to prepare the stock solution shortly before each experiment. Solutions were mixed with NGM agar at the required concentration just before pouring to 6 cm plates. Plates were allowed to dry at room temperature for two days and then spotted with *Novosphingobium* L76.

#### The TEI models

Systems of ordinary differential equations, representing different models of TEI dynamics, were numerically integrated in Python 3.12 using SciPy 1.15.3.^41^

### Quantification and statistical analysis

The probability of assuming the predatory mouth form by the adult nematodes was calculated using the following hierarchical bayesian model (Figure S1A):

(Equation 7) $$\begin{array}{cc} y_{i} & ∼\text{Binomial}\left(N,\theta_{i}\right)\\ \theta_{i} & ={\text{logit}}^{-1}\left(\eta_{i}\right)\\ \eta_{i} & =\mu +z_{i}\sigma \\ z_{i} & ∼\text{Normal}\left(0,1\right)\\ \mu & ∼\text{Normal}\left(0,2\right)\\ \sigma & ∼\text{HalfNormal}\left(2\right) \end{array}$$

Using the inferred parameters, the predictive probability for the development of the predatory mouth form is calculated:

(Equation 8) $$\begin{array}{cc} \tilde{z} & ∼\text{Normal}\left(0,1\right)\\ \tilde{\theta } & ={\text{logit}}^{-1}\left(\mu +\tilde{z}\sigma \right). \end{array}$$

The 95% highest density interval (HDI) for $\tilde{\theta }$ is used to visualize the inferred probability of developing the Eu mouth form.

The effect of TEI is quantified using the risk difference (RD) between generation i and the first generation following reversal as baseline:

(Equation 9) $$\text{RD}={\tilde{\theta }}_{i}-{\tilde{\theta }}_{\text{baseline}}.$$

The rate of decline in epigenetic memory was estimates using a bayesian model. Given the following likelihood function for the mouth form of naive worms (P0):

(Equation 10) $$\begin{array}{cc} z_{i}^{\text{P0}} & ∼N\left(0,1\right)\\ \text{logit}\left(\theta^{{\text{P0}}_{i}}\right) & =\mu_{\text{P0}}+z_{i}^{\text{P0}}\sigma_{\text{P0}}\\ y_{i}^{\text{P0}} & ∼\text{Binomial}\left(n,\theta_{i}^{\text{P0}}\right), \end{array}$$

and the likelihood function for the mouth form for generation t during reversal

(Equation 11) $$\begin{array}{cc} z_{t} & ∼N\left(0,1\right)\\ \text{logit}\left(\theta_{t}\right) & =\mu_{t}+z_{t}\sigma \\ y_{t} & ∼\text{Binomial}\left(n,\theta_{t}\right), \end{array}$$

we model the loss of epigenetic memory as exponential decay:

(Equation 12) $$\mu_{t}=\mu_{{\text{P0}}_{i}}+\left(\mu_{R_{1}}-\mu_{{\text{P0}}_{i}}\right){\left(1-\kappa \right)}^{t},$$

where κ represent the rate at which the epigenetic memory – relative to the first generation grown on the non-inducing environment following induction or superinduction $\left(R_{1}\right)$ – declines (Figure S1B).

The model was fitted to the laboratory measurements using PyMC^39^ using Python 3.12 with Numpy 2.3.0.^40^ We ensured that the estimated values were stable using common convergence diagnostics^42^ (Figure S2).
