# Supplementary material for: The logic of transgenerational inheritance in a nematode worm
Source: iScience. 2026 Sep 3;29(9):117401. doi: 10.1016/j.isci.2026.117401 (PMC13571746; doi:10.1016/j.isci.2026.117401)
Supplement: Supplementary file 1 [file mmc1.pdf]

iScience, Volume *29*

Supplemental information

The logic of transgenerational inheritance in a nematode worm

Shiela Pearl Quiobe, Ata Kalirad, and Ralf J. Sommer

Supplementary information

The logic of transgenerational inheritance of cannibalism

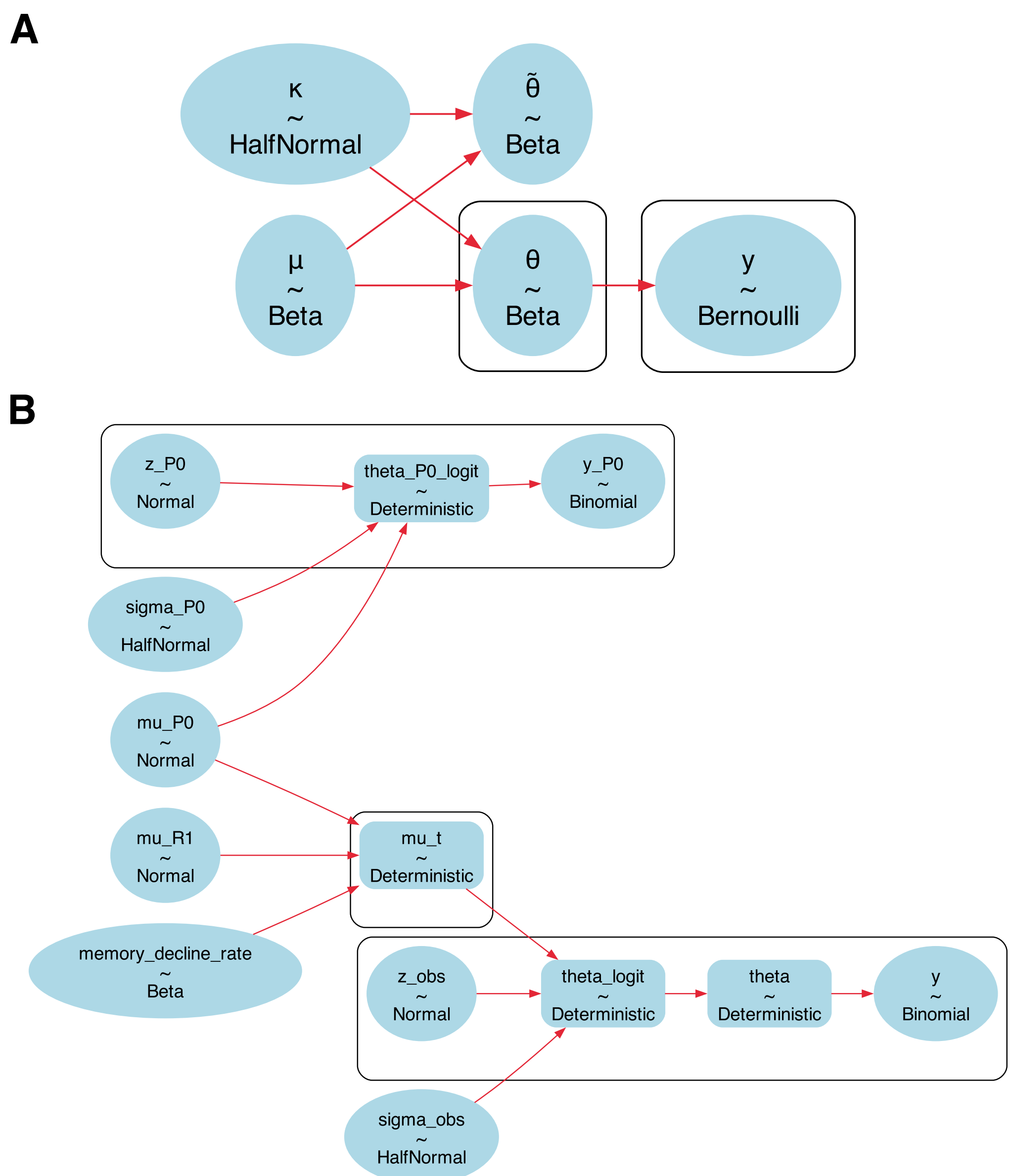

Figure S1

Figure S1. Structure of the Bayesian hierarchical models used to infer the probability of the predatory mouth form and the rate of decline in epigenetic memory.

(A) The structure of the bayesian hierarchical model constructed to infer the probability of developing the predatory mouth form. (equations 7 and 8). The number of predatory adults per plate is used as the input, resulting in inference of θ_i for plate i.
(B) The structure of the bayesian hierarchical model constructed to infer the rate of decline in epigenetic memory (equations 10–12).

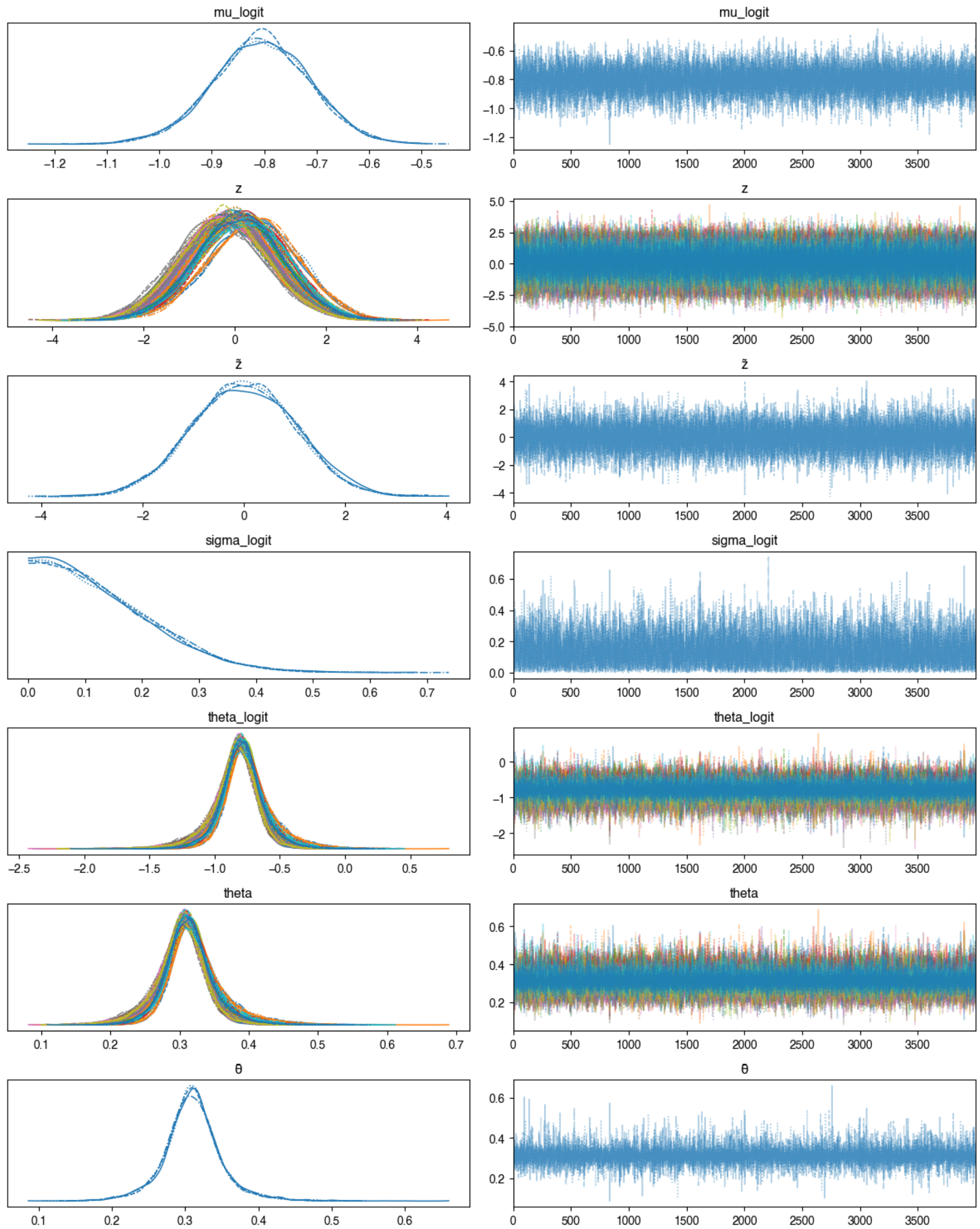
mu_logit
mu_logit
z
z
z̃
z̃
sigma_logit
sigma_logit
theta_logit
theta_logit
theta
theta
θ
θ

Figure S2

Figure S2. Representative posterior density and trace plots illustrating convergence of the Bayesian model.

An example of the typical output of the model, visualized using ArviZ 0.21., which represents the posterior density plots (left column) for chains, including for parameters that are estimated on the plate level, and the trace plots (right columns) indicating the convergence of the model.

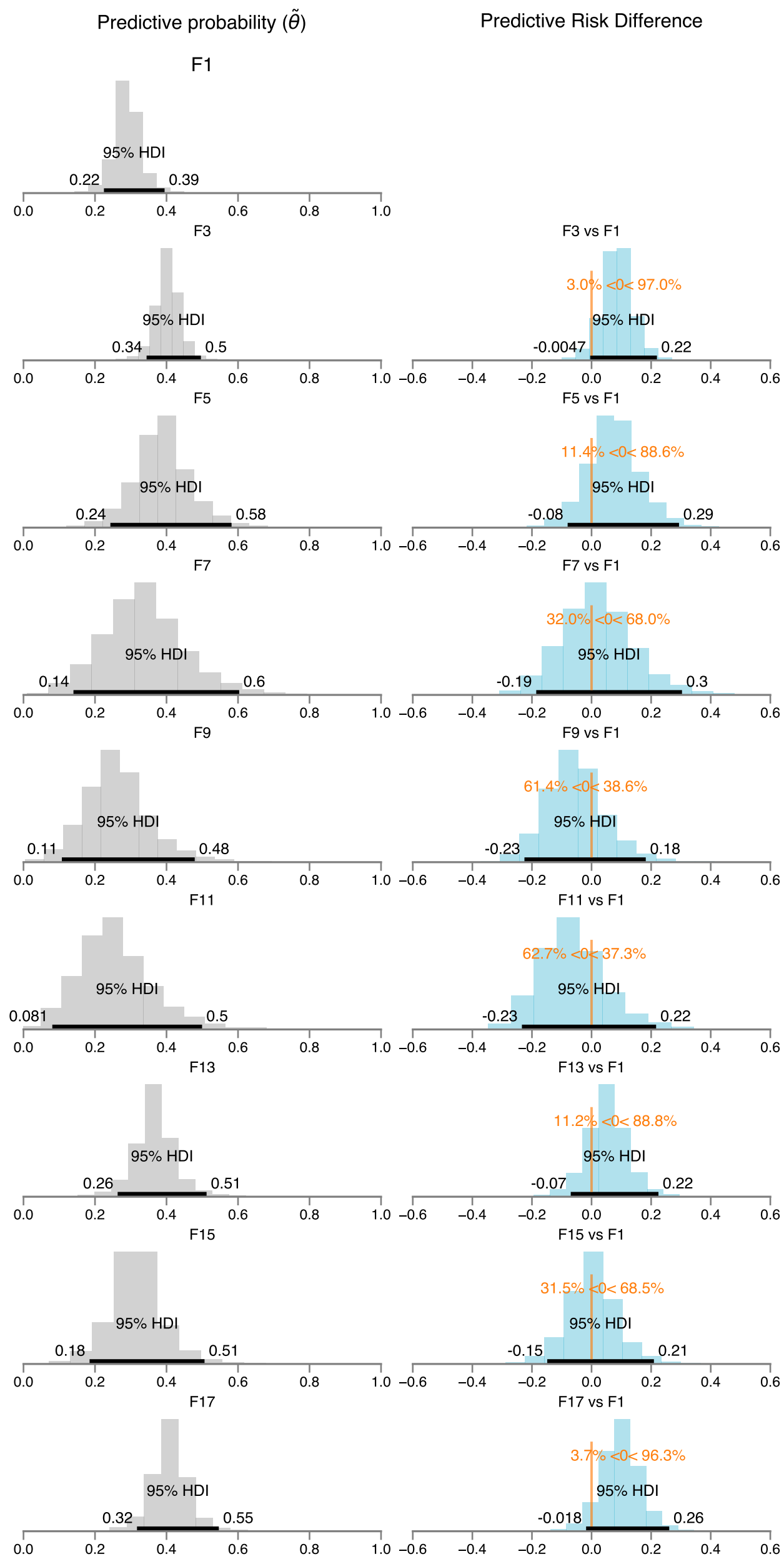
Predictive probability ($\tilde{\theta}$)
Predictive Risk Difference
F1
95% HDI
0.22 0.39
F3
95% HDI
0.34 0.5
F3 vs F1
3.0% <0< 97.0%
95% HDI
-0.0047 0.22
F5
95% HDI
0.24 0.58
F5 vs F1
11.4% <0< 88.6%
95% HDI
-0.08 0.29
F7
95% HDI
0.14 0.6
F7 vs F1
32.0% <0< 68.0%
95% HDI
-0.19 0.3
F9
95% HDI
0.11 0.48
F9 vs F1
61.4% <0< 38.6%
95% HDI
-0.23 0.18
F11
95% HDI
0.081 0.5
F11 vs F1
62.7% <0< 37.3%
95% HDI
-0.23 0.22
F13
95% HDI
0.26 0.51
F13 vs F1
11.2% <0< 88.8%
95% HDI
-0.07 0.22
F15
95% HDI
0.18 0.51
F15 vs F1
31.5% <0< 68.5%
95% HDI
-0.15 0.21
F17
95% HDI
0.32 0.55
F17 vs F1
3.7% <0< 96.3%
95% HDI
-0.018 0.26

Figure S3

Figure S3. Expanded posterior predictive and risk-difference analysis for the alternative induction experiment with $n = 1$ (Figure 2B).

The left column visualizes the posterior predictive of $\tilde{\theta}$ for each generation Fi. The right column indicates the predictive RD, calculated by subtracting the inferred $\tilde{\theta}$ for each generation form that of the first generation (F1). The orange text indicates the percentage of the distribution for RD that is lesser or greater than zero. The black horizontal bar indicates the 95% HDI for each parameter.

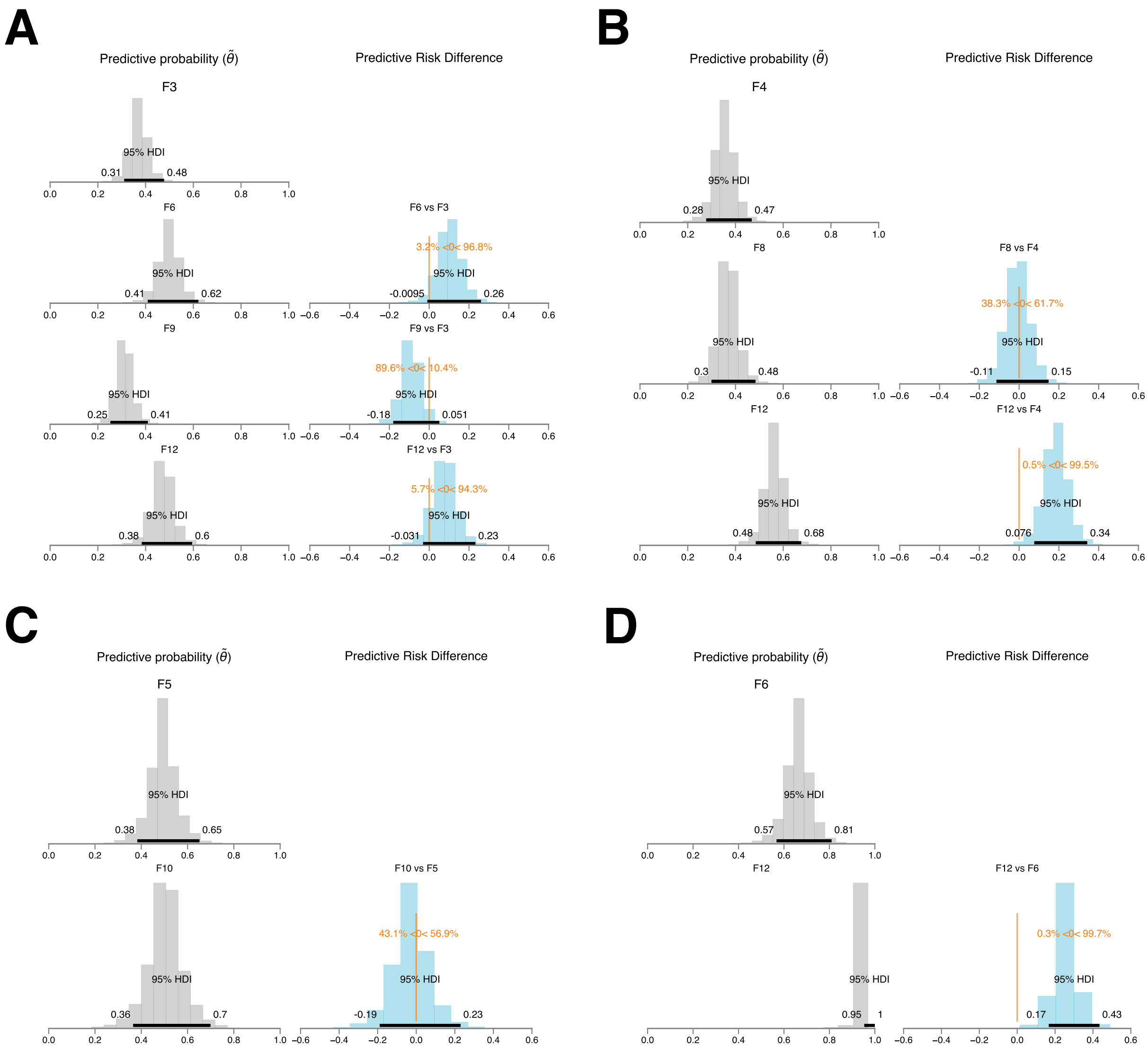
A
Predictive probability ($\tilde{\theta}$)
Predictive Risk Difference
F3
F6
F9
F12
F6 vs F3
F9 vs F3
F12 vs F3
95% HDI
B
F4
F8
F12
F8 vs F4
F12 vs F4
C
F5
F10
F10 vs F5
D
F6
F12
F12 vs F6

Figure S4

Figure S4. Expanded posterior predictive and risk-difference analysis for the alternative induction experiments with $n = 2$, 3, and 4 (Figure 2C–F).

The expanded analysis for Figure 2C **(A)**, Figure 2D **(B)**, Figure 2E **(C)**, and Figure 2F **(D)**. In each subplot, the left column visualizes the posterior predictive of $\tilde{\theta}$ for each generation Fi. The right column indicates the predictive RD, calculated by subtracting the inferred $\tilde{\theta}$ for each generation form that of the first generation (F1). The orange text indicates the percentage of the distribution for RD that is lesser or greater than zero. The black horizontal bar indicates the 95% HDI for each parameter.

Table S1. Parameters used in three models of TEI (Figure 1C)

Parameter	Value	Description
**Shared Regulatory Core**		
α_X, β_X	2.0, 1.0	Production and decay rates for species X.
α_Y, β_Y	1.5, 0.2	Production and decay rates for species Y.
K_{XY}, n_{XY}	0.3, 3.0	Hill threshold and cooperativity for X repressing Y.
α_Z, β_Z	1.5, 0.5	Baseline production and decay rates for output Z.
τ_R, k_R	25.0, 0.3	Resource recovery timescale and depletion rate.
**Acclimation TEI**		
k_A	0.2	Accumulation rate of inhibitor A during trigger.
τ_A	30.0	Recovery timescale for the acclimated state.
γ_A	2.0	Strength of A-mediated inhibition on Z production.
**Sensitization TEI**		
k_S	0.2	Accumulation rate of sensitizer S during trigger.
τ_S	40.0	Persistence timescale for the sensitized state.
c_S	3.0	Factor by which S reduces the degradation of Z.

Table S2. Sample sizes used in each experiment. For Figure 2, N denotes the number of worms assayed per generation; for Figures 3–5, N denotes the total number of worms assayed.

Figure	B	C	D	E	F	G
**2**	340–620[a]	500[b]	520/340/420/400/420[c]	480	540	—
**3**	880	960	1,100	1,200	1,320	1,440
**4**	1,540	1,680	—	—	—	—
**5**	1,980	2,280	—	—	—	—

[a] Range across generations.
[b] Per generation; except generation 6 ($N = 420$) and generation 12 ($N = 300$).
[c] Generations 1–3 / generation 4 / generations 5–7 / generation 8 / remaining generations.
